# Does human milk composition predict later risk of obesity? A systematic review

**DOI:** 10.1186/s40795-023-00742-9

**Published:** 2023-07-20

**Authors:** Mayara Vieira Queiroz De Paula, Maude Grant, Julie Lanigan, Atul Singhal

**Affiliations:** 1 Childhood Nutrition Research Centre, UCL Great Ormond Street Institute of Child Health, London, UK; 2 Nestlé Nutrition, Société des Produits Nestlé, Vevey, Switzerland

**Keywords:** Breastmilk, Breastfeeding, Human milk composition, Obesity, Obesity risk, Body composition, Infants, Children, Growth

## Abstract

**Background:**

Possible mechanisms behind the association of breastfeeding with a lower risk of later obesity are unknown but one possibility is the unique composition of human milk. Here, we systematically reviewed the evidence linking breast-milk macronutrient and hormonal composition with later obesity.

**Methods:**

We searched 7 databases for studies that included infants predominantly breast-fed for the first 3 months and which analysed associations between a measure of breast-milk composition and later (> 6 months) measures of obesity or body composition.

**Results:**

47 publications were identified for full-text screening, of which 10 were eligible and only 3 found significant associations. Higher leptin concentration in breast milk at age 1 month was associated with lower infant BMI at 12, 18 and 24 months of age (1 study). Higher breast-milk adiponectin concentration at 6 weeks and 4 months were associated with adiposity at age 12 and 24 months (1 study). In 1 study, breast-milk carbohydrate content was positively associated, and fat content negatively associated, with adiposity at age 12 months. No significant associations were found between other hormones or macronutrients in human milk and later risk of obesity or body composition.

**Conclusions:**

The evidence linking breast-milk composition with later obesity was inconsistent and confined to single, individual studies. Our review highlights the methodological limitations of previous studies and the need for further research in this area.

**Supplementary Information:**

The online version contains supplementary material available at 10.1186/s40795-023-00742-9.

## Background

Obesity in children is a major global public health issue with an estimated 16% of children and adolescents, and 38.2 million children under 5 years, living with overweight or obesity globally [1]. Children who have overweight and obesity are more likely to stay overweight and obese into adulthood and are at higher risk of developing non-communicable diseases such as cardiovascular disease and type 2 diabetes at an earlier age than in children without obesity. While genetic, environmental and lifestyle factors are the major determinants [2], there is compelling evidence that factors acting during a critical window in the ‘first 1000 days’, the period from conception to 2 years of age, also influence or ‘programme’ the risk of later obesity [3], a concept known as the developmental origins of health and disease hypothesis [4]. Early postnatal nutrition has shown to have strong programming effects with several systematic reviews finding a lower risk of obesity in breast-fed compared to formula-fed infants [5, 6]. However, whether breastfeeding is causally related to a lower risk of obesity is controversial, partly because the potential mechanisms remain poorly understood, and because of the possibility of confounding factors (e.g. maternal education) influencing both a mother’s decision to breast-feed and lifestyle risk factors for developing obesity [7–9].

The protective effect of breastfeeding against obesity has been suggested to be partly explained by its unique composition [10, 11]. For example, a lower concentration of protein in human milk compared to formula is suggested to lead to a slower pattern of weight gain and, in turn, reduce the later risk of obesity, as described in the postnatal growth acceleration hypothesis [12]. Higher protein intake in infant formula could increase concentrations of insulin and insulin-like growth factor 1 (IGF-1), stimulating both accelerated growth and adipogenesis as described in the early protein hypothesis [13, 14]. Other factors found in human milk that are associated with infant growth and later obesity include human milk oligosaccharides (HMO), long-chain polyunsaturated fatty acids (LCPUFA), and hormones such as leptin, insulin, ghrelin, and adiponectin. However, whether these factors are causally related to a lower risk of obesity in breast-fed infants remains unknown [15–17].

The potential role of confounding factors in explaining the lower risk of obesity in breast-fed versus formula-fed infants may be partly addressed by investigating associations between human milk composition and later obesity *within a predominantly breast-fed population*. In one such study, Prentice et al. showed that a lower concentration of fat in human milk at 4–8 weeks of age was associated with greater gains in BMI and adiposity in the first year, whilst higher carbohydrates and protein concentrations were both associated with a higher BMI at age 12 months, with the same pattern of associations observed in the sub-analysis consisting of infants exclusively breastfed [18]. In contrast, Gridneva et al. (2018) found that the concentration of protein in human milk was not associated with body composition at age 12 months [19]. Thus, whether human milk composition impacts later obesity and body composition remains unknown.

Previous reviews have investigated the effects of human milk hormones or macronutrients on short-term infant growth and body composition but not on later risk of obesity [20–22]. As such, the aim of this systematic review was to synthesize the existing literature on associations between human milk nutritional composition and later measures of obesity, obesity risk or body composition beyond infancy. Findings from such a review could help to better understand the mechanisms for the beneficial effects of breastfeeding on later health and inform targeted prevention strategies for obesity (e.g., prevention of overfeeding) in both breast-fed and formula-fed infants.

## Methods

This systematic review was reported in line with the Preferred Reporting Items for Systematic Reviews and Meta-Analysis statement (PRISMA) [23]. The PRISMA 2020 checklist shows this (see Additional File 1). The protocol was registered in PROSPERO (registration number: CRD42020193664).

### Eligibility criteria

Only studies published in English were included as this was the native language of the reviewers. All cohort studies including longitudinal, prospective cohort, birth cohorts or pilot studies were eligible. Randomized controlled trials (RCTs) and quasi-randomized controlled trials of mothers who received nutritional supplementation (e.g., long-chain polyunsaturated fatty acids [LCPUFA], or human milk oligosaccharides [HMO]) before and during lactation, and where there was a subsequent measure of human milk composition and later risk of obesity, or body composition were included. Data from RCTs were treated as a cohort because only associations between human milk composition and later measures of obesity or body composition were of interest. RCTs which supplemented micronutrients were excluded because these nutrients have not been hypothesized to be associated with a later risk of obesity in breast-fed or formula-fed infants. Studies where only the abstract was available were excluded as the authors would not be able to extract the relevant data.

Studies in which most (> 75%) infants were exclusively or predominantly breast-fed (defined as receiving < 125 ml formula/day) for the first three months were eligible. Populations predominantly breast–feeding (as opposed to exclusively breast-fed) were chosen as an eligibility criterion since in many populations mixed feeding is common whereas exclusive breast-feeding is relatively rare. Due to the scarcity of studies that included exclusive or predominantly breastfed infants with a measure of human milk composition, and in order not to exclude potentially relevant studies, no restrictions were made on study country or setting or participant ethnicity. Excluded studies included animal models, infants < 37 weeks’ gestation, infants born small for gestation (defined as birthweight < 2 SD below the mean, or < 10th percentile according to the growth charts used), and infants with clinical conditions or chronic disease that could influence patterns of growth.

Eligible studies were those which measured human milk components expressed as a whole or its constituent parts including energy, protein (total protein and/or amino acids), lipids (total lipids and/or triglycerides, fatty acids), carbohydrates (total carbohydrates and/or glucose, lactose, fructose, HMO), LCPUFA of the omega-6 (n-6) series (linoleic acid and arachidonic acid and omega-3 (n-3) series (α-linolenic acid, eicosapentaenoic acid, and docosahexaenoic acid), leptin, insulin, adiponectin, ghrelin, and cortisol. Only studies analysing mature milk, and not colostrum were considered eligible because colostrum composition is highly variable over time, and has relatively low concentrations of lactose, lipids, and energy, in line with its mainly immunological rather than nutritional function [24].

The primary outcome was an association between a measure of obesity or obesity risk captured ≥ 6 months (± 2 weeks) after the associated measure of human milk composition. A gap of 6 months between exposure and outcome was chosen arbitrarily to assess ‘later’ or ‘programming’ effects of breast milk composition on obesity. Acceptable measures of obesity or obesity risk included body mass index (BMI), BMI percentile, and BMI z-score in children < 18 years, and BMI in adults, obtained at a specific time point or analysed as the change between two time points. Since there are different growth charts and BMI cut-offs for obesity, all definitions for obesity based on any growth chart were accepted. The secondary outcome was associations between a measure of body composition captured ≥ 6 months (± 2 weeks) after the associated measure of human milk composition. Measures of body composition, reported either as an absolute value at a specific time point, or as a change between two time points, were acceptable. These measures included skinfold thickness (any number of sites), or any more sophisticated techniques to quantify fat and fat free mass (e.g., deuterium dilution), expressed as an absolute amount or as in any index (e.g., fat mass index).

### Information sources

Studies were identified by searching electronic databases with initially no restrictions applied on language, date, or publication status. The electronic databases searched included Cochrane Central Register of Controlled Trials (CENTRAL; current issue) part of the Cochrane Library, MEDLINE Ovid (from 1946), Embase Ovid (from 1974), Maternity and Infant Care (MIDIRS) (from 1971), PubMed (from 1982), Web of Science Core Collection Clarivate (1990 onwards) and SCOPUS Elsevier (1979 onwards).

### Search strategy

The search strategy included database-specific terms and medical subject headings (MeSH) with Boolean operators, truncations, wildcards, and synonyms of terms relating to human milk, obesity, anthropometry, hormones, and macronutrients. Searches were limited to human studies and excluded measures of cognition, infection, complementary feeding, and breast-feeding duration. In addition, reference lists of studies in full-text review were searched to identify possible omissions. The search was updated on 26 April 2023. Full search results are available (see Additional File 2).

### Selection strategy

Studies retrieved from the search were uploaded to the reference management system EndNote X9 3.3 before being transferred to the Covidence system for duplicate removal and screening. Eligibility assessment was conducted by two independent reviewers (MDP and MG) in a two-stage process. Titles and abstracts were screened first and studies that met inclusion criteria underwent full-text review. Disagreements between reviewers were resolved through consultation with a third reviewer (JL).

### Data collection

Data to be extracted was agreed a priori by the research team. Two reviewers (MDP and MG) extracted data independently and any inconsistencies and disagreements were resolved through mutual discussion. Where necessary, authors were contacted for clarification (on 2 occasions and with one response). Extracted data included author name, date of publication, source of study funding and conflicts of interest, study design, aim, duration, population, feeding type and duration, timing and type of human milk component, obesity and anthropometric measures, results of associations and confounders.

### Quality assessment

Two independent reviewers (MDP and MG) used the revised Downs and Black Quality Index score system, known to be a valid and reliable tool for assessing bias in randomized and observational studies to assess the quality of individual publications [25]. Item 27 relating to statistical power was modified so that a score of 1 was given where a power calculation was performed and 0 if this was not the case [26]. The maximum total quality score using this system was 28, a score of ≥ 26 was considered excellent, a score between 20 – 25 was considered good, 15 – 19 was considered fair, and a score ≤ 14 was considered low [27]. The quality assessment tool provided an overall quality score based on four assessed domains including reporting, external validity, internal validity bias and internal validity-confounding [25]. Any disagreements were resolved through discussion.

## Results

### Study selection

**Fig. 1:**
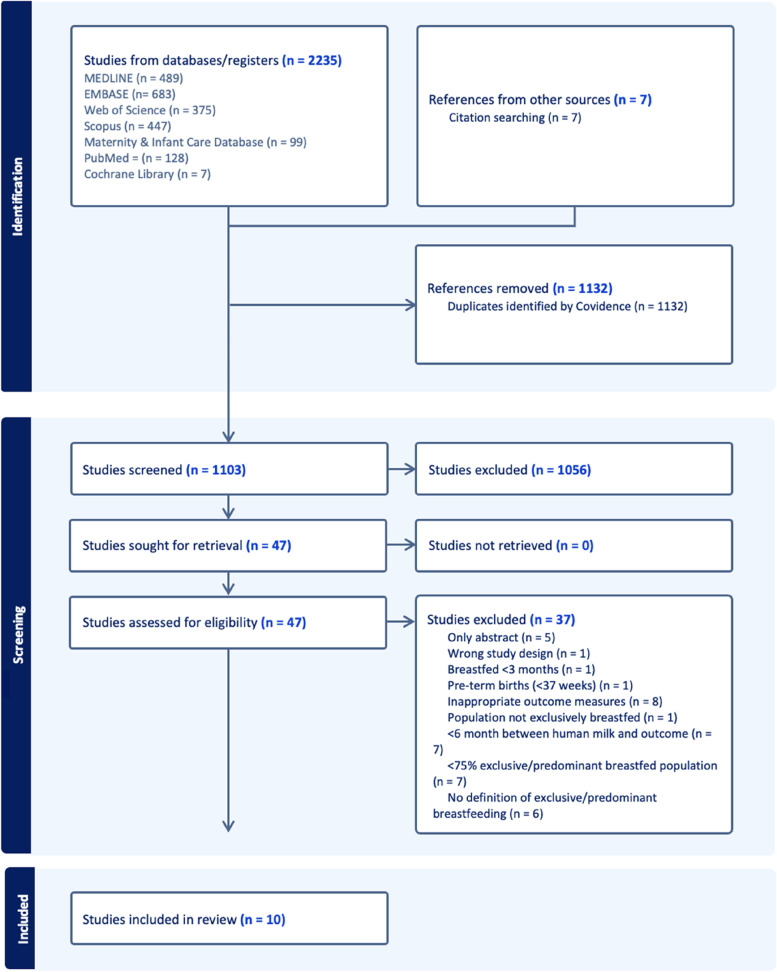
Preferred Reporting Items for Systematic Reviews and Meta-Analysis (PRISMA) flow diagram summarizing the process of study screening and reasons for exclusion

We identified 2235 papers, of which 1103 were screened following the removal of duplicates, with the remaining 47 eligible for full-text assessment. In total, 10 papers [18, 19, 28–35] were included in the narrative synthesis from 9 original studies [18, 19, 28–31, 33–35]. Meyer et al. [32] was a later follow-up from the same cohort reported in Brunner et al. [28], and three of the papers were original analysis involving the same population [19, 30, 31]. See Fig. 1 for PRISMA flow diagram.

### Data synthesis

Once the data from eligible studies were extracted it became clear that it was not possible to conduct a meta-analysis on the relationship between human milk composition and later obesity due to the highly heterogenic data for both measures of human milk composition and obesity. As such, data was synthesized narratively and presented in tabular format.

### Study characteristics

All included studies were conducted in high-income countries, and most were of cohort design [18, 19, 29–31, 33–35]. Two studies were follow-ups of a previously conducted RCT [28, 32]. Human milk components analysed included concentrations of leptin [28, 30, 32, 33], adiponectin [28, 30, 32], carbohydrates [18, 29, 31, 34, 35], protein [18, 19, 29, 34, 35], fat [18, 29, 34, 35], energy [18, 34], insulin, principal component analysis (PCA) of fatty acids, and LCPUFA n-6 to n-3 ratio [29]. There were no eligible studies found for ghrelin, cortisol, or HMO concentrations. Data for all eligible studies including the methods for collection and analysis of human milk samples and measurements of obesity and/or body composition are summarised (see Additional file 3).

### Quality assessment

Quality assessment scores are found in Table 1. Included papers were of low to fair quality with scores ranging from 7 to 18 (mean, SD: 14.2, 3.1). The average score for the reporting section (maximum 11) was mediocre, with a mean score of 7 ± 1.25. The external validity rating was poor across most studies with a mean score of 0.8 ± 1.1 out of a maximum of 3. The internal validity bias rating was average, (with a mean score of 3.9 ± 0.9 out of a maximum of 7) because, as expected, none of the studies blinded study subjects or researchers to the exposure (human milk composition). In assessing the internal validity—confounding, the mean score was 2.1 ± 0.8 from a maximum score of 6. Only 6 of the chosen publications adjusted for confounding variables in the analysis [18, 28, 29, 32, 34, 35]. Lastly, only 4 studies conducted a power calculation, which scored an extra 1 point [19, 30, 31, 35].

**Table 1:** Risk of bias assessment

Included papers	Reporting	External Validity	Internal Validity—Bias	Internal Validity – Confounding	Power	Score	Rating
Brunner et al. 2015 [28]	8	2	5	3	0	18	
De Fluiter et al. 2021 [34]	8	0	3	2	0	13	
Ellsworth et al. 2020 [29]	6	1	5	2	0	14	
Gridneva et al. 2018 [30]	7	0	4	2	1	14	
Gridneva et al. 2018 [19]	7	0	4	2	1	14	
Gridneva et al. 2019 [31]	7	0	4	2	1	14	
Meyer et al. 2017 [32]	8	2	5	3	0	18	
Miralles et al. 2006 [33]	4	0	3	0	0	7	
Olga et al. 2022 [35]	8	0	2	2	1	13	
Prentice et al. 2016 [18]	7	3	4	3	0	17	

Results from risk of bias assessment using the modified Downs and Black checklist. Scores between 15 – 19 was considered fair (  ), and scores ≤ 14 was considered low (  )

### Synthesis of results

Results of associations between human milk composition and later measures of obesity were summarised (see Additional file 4). Eight out of the 10 selected papers reported associations between human milk composition and later measures of BMI [19, 28–33, 35]. Of these, only Miralles et al. [33] reported an association between higher concentration of leptin in human milk at 1 month of age and a lower BMI at age 12, 18, and 24 months. In contrast, Brunner et al. [28] found no significant associations between either human milk leptin concentration at age 4 months, or adiponectin concentration at age 6 weeks and infant BMI at 12 or 24 months of age. Likewise, Gridneva et al. [30] found no significant association between calculated daily intakes of leptin between 2 and 5 months of age and change in infant BMI (kg/m^2^) between 2 and 12 months, or between 5 and 12 months. Meyer et al. [32] also reported no significant associations between both 6-week leptin or adiponectin concentrations and BMI percentile at age 3–5 years. No significant associations were found between other hormones or macronutrients in human milk and later risk of obesity [19, 29, 31, 35].

Results of associations between human milk composition and later measures of body composition were summarised (see Additional file 5). Eight of the 10 chosen papers investigated associations between human milk composition and later body composition [18, 19, 28, 30–32, 34, 35]. Prentice et al. [18] found that higher carbohydrate content in human milk (expressed as percentage of total energy) at age 4–8 weeks was associated with adiposity at 12 months of age while a higher percentage of fat in human milk at 4–8 weeks of age was associated with lower adiposity at age 12 months. However, there were no significant associations between the protein or energy content of human milk and later adiposity [18]. Equally, Olga et al. found no significant association with carbohydrate, protein or fat at 6 weeks and sum of four skinfold SDS at 12 months in infants exclusively breast fed for the first 6 months [35]. In terms of hormones, Brunner et al. [28] reported that a higher concentration of adiponectin in human milk when the infant was aged 6 weeks was associated with higher fat mass (expressed as sum of four skinfolds, total fat mass and percentage body fat) at age 12 months, but not at 24 months of age. The concentration of adiponectin in human milk at 4 months of age was also associated with greater fat mass (expressed as sum of skinfolds at four sites, absolute and percentage body fat) at 24 months of age in the adjusted analyses. In contrast, Meyer et al. and Gridneva et al. [30, 32] found no association between human milk adiponectin concentration measured at several time points and adiposity between 2 and 12 months, and 5 and 12 months [30], or later in childhood at 3, 4, 5 years of age [32]. No significant associations were reported for the concentration of leptin in human milk at age 4 months and measures of body composition at 12 or 24 months of age [28]. No other publications reported significant associations between other hormones [30, 32] or macronutrient concentrations and later body composition after statistical adjustment for multiple testing [19, 31, 34, 35]. Few eligible studies adjusted for confounding factors in the analysis [18, 28, 32, 34, 35]. However, Brunner et al. confirmed that the association between adiponectin and adiposity measured at 1 year remained significant after adjustment for maternal pre-pregnancy BMI, gestational weight gain, sex, and type of infant feeding (exclusively or partially breastfed) [28].

## Discussion

To the best of our knowledge, this is the first systematic synthesis of the literature on longitudinal associations between breast-milk composition and later measures of obesity and body composition. Specifically, in contrast to individual studies, our systematic synthesis found little evidence of consistent associations between energy, macronutrient and hormonal content of human milk and measures of later obesity or adiposity. The review highlights the inconsistency of the evidence linking breast milk composition and later risk of obesity, the need to better understand mechanisms supporting the protective effects of breastfeeding on later obesity, and the need for further research in this area.

Previously, hormones such as adiponectin and leptin which play key roles in energy homeostasis and satiety (and are found in human milk but absent from formula) have been hypothesized to contribute to the protective effect of breast feeding against obesity [36, 37]. Leptin receptors have been identified in the gastric and absorptive cells of human and animal models, suggesting that leptin can pass from breast-milk to the bloodstream [38]. Therefore, leptin ingested in breast-milk has been proposed to influence infant energy regulation by upregulating hypothalamic neurons that reduce appetite and suppressing those that increase food intake [36]. However, of the 4 published studies that investigated the association between breast-milk leptin concentration and later obesity or body composition [28, 30, 32, 33], only Miralles et al. [33] demonstrated a negative association with later BMI in the first 24 months suggesting that this hypothesis remains uncertain. Similarly, only 1 of 3 studies found an association between human milk adiponectin concentration and later obesity or body composition [28, 30, 32]. In the only positive study, adiponectin concentration at age 6 weeks was associated with greater adiposity at age 12 months, and concentrations at age 4 months were associated with greater adiposity at 24 months [28], but not later in childhood (3–5 years of age) suggesting that any association was likely to be short-term [32]. The reasons for these inconsistent findings are uncertain, but could include the lack of understanding on how leptin and adiponectin in breast-milk reach the infant’s circulation in a biologically active form [39]. Lastly, of the papers investigating hormones, only one study investigated the concentration of insulin in human milk but reported no significant associations with later BMI [29]. Overall, the limited number of studies found in this review, and lack of consistent associations, casts doubt on the hypothesis that hormones found in breast-milk such as leptin, adiponectin or insulin influence the later risk of obesity, a finding in agreement with a recent systematic review that also found conflicting evidence on a role for human milk hormones on infant growth [40].

Surprisingly, we found limited evidence of any associations between breast-milk macronutrient composition and later obesity risk or body composition [18, 19, 29, 31, 34, 35]. Only one study [18] found that a higher percentage of fat in breast-milk was associated with lower adiposity at age 12 months while a higher percentage of carbohydrate was associated with greater adiposity at 12 months, independent of birthweight, gestational age, infant sex, nutrition type and HM storage time [18]. Possible explanations for these finding are unclear, but the authors suggested that a higher fat content in human milk may have greater satiating effect that help to better regulate the development of longer-term appetite [41]. Lastly, despite evidence for effects of LCPUFA on adipogenesis [42, 43], we found no evidence of any association between the LCPUFA content of human milk and risk of later obesity, even though LCPUFAs may have beneficial effects for other outcomes such as neurodevelopment.

Perhaps the most surprising finding was the limited evidence for any association between breast milk protein content and later obesity or body composition [18, 19, 29, 34, 35]. The study by Prentice et al. found that breast-milk protein concentration was associated with later risk of obesity in the predominantly breast-fed but not in exclusively breast-fed infants, suggesting that protein in human milk has different effects on programming of obesity than protein in formula possibly because of differences in protein quality. Due to the strict selection criteria for this review (> 75% of infant participants exclusively or predominantly breast-fed [defined as receiving < 125 ml formula/day]), only the results from the sub-analysis of exclusively breast-fed infants were included. A high protein intake in early life has been consistently associated with greater risk of obesity [14, 44] and adiposity later in life [13] in epidemiological studies [45] and RCTs in formula–fed infants [5, 14, 44, 46]. There is also anecdotal evidence in exclusively breast–fed infants that a high protein concentration in breast-milk (mean 1.25 g/dl compared to the reference of 0.8 g/dl) is associated with excessive weight gain in infancy and a higher risk of later obesity [47, 48]. However, only 5 studies have investigated associations of human milk protein concentration with risk of later obesity [18, 19, 29, 34, 35] and hence there is insufficient evidence to suggest that the early protein hypothesis is applicable to exclusively or predominantly breastfed infants.

A major strength of this review is the comprehensive and systematic assessment of the data linking human milk composition and longitudinal risk of obesity and adiposity in infancy. Another strength was the independent completion of study screening, selection, bias assessment, and data extraction by two reviewers, thereby reducing bias. Studies investigating cross-sectional associations between human milk composition and measures of obesity were specifically excluded to focus on the factors in human milk which could explain the longer-term (or ‘programming’) protective effects of breast feeding on risk of developing obesity.

This systematic review has several limitations. First, the studies used varying methods for assessing human milk composition, obesity, and body composition. Human milk components were expressed using different parameters (e.g., concentrations, percentages, daily intakes) which made comparisons between studies difficult. As such, due to this heterogeneity, it was not possible to standardise the exposures and outcomes of interest and conduct a meta-analysis. Second, most studies in this area were generally of low quality with none rated as ‘good’ or above. Third, widely variable and non-standardised methods of collecting and handling human milk samples means that human milk composition data is likely to have been affected by factors such as circadian rhythms and infant’s age. For instance, protein content decreases over the first few months following birth [49], whilst fat content of human milk is highly variable and influenced by time of day and increases throughout the feed as the breast is emptied [50]. In addition, only the Gridneva et al. studies and most recently Olga et al. considered volume of breast-milk intake, which would affect the total amount of human milk components ingested [19, 30, 31, 35]. Human milk composition is also affected by the total volume of milk. For instance, in the DARLING study, protein and lipid concentrations in human milk were lower with a higher volume of milk, whilst lactose concentration was positively related to human milk volume [41]. Fourth, only 6 of 10 studies accounted for potential confounding factors in data analyses [18, 28, 29, 32, 34, 35], an important omission given that both human milk composition and obesity risk may be influenced by maternal anthropometry and socio-demographic factors. Finally, only a small number of studies were available to review. This is partly because many studies did not define or report infant feeding practices, or the sample included a significant proportion of formula-fed infants. However, using a strict inclusion criterion of > 75% breast feeding was felt to be important because of the well-known association of formula feeding with later obesity [5, 14, 44, 46].

## Conclusions

Overall, this systematic review found insufficient evidence to support the hypothesis that human milk composition is associated with later risk of obesity or body composition. The review highlights important methodological limitations of previous research and so we recommend that the following be considered in future studies in this area. First, standardized protocols for the collection and analysis of human milk samples are essential to ensure representative samples that can be compared across studies. Second, measurement of milk volume intake will be critical to assess the absolute intake of nutrients and so may provide a better mechanistic insight into the link between human milk composition and later obesity. Third, data analysis should control for known confounding variables that could affect human milk composition and measures of obesity. Future research using these recommendations could help identify infants more susceptible to developing later obesity, optimize the composition of infant formula, and inform strategies for the prevention of obesity in both breastfed and formula-fed infants.

## Supplementary Information

**Additional file 1. **PRISMA 2020 Checklist. PRISMA 2020 checklist with page numbers for this systematic review.

**Additional file 2. **Literature Search. Summary of all search terms and results.

**Additional file 3. **Study Characteristics. Summary of all included study characteristics.

**Additional file 4. **Obesity Study Findings. Summary of all included studies with a measure of human milk composition and obesity. Where significant findings were found, the results were displayed.

**Additional file 5. **Body Composition Study Findings. Summary of all included studies with a measure of human milk composition and measures of body composition. Where significant findings were found, the results were displayed. 

## Data Availability

Data sharing is not applicable to this article as no datasets were generated or analysed during the current study.

## Abbreviations

2SF: 2-Skinfolds
4SF: 4-Skinfolds
BC: Body composition
BIS: Bio impedance spectroscopy
BMI: Body mass index
CDI: Calculated daily intake
CI: Confidence interval
EBF: Exclusively breastfed
EP: Electric pump
F: Female
FFM: Fat-free mass
FFMI: Fat-free mass index
FM: Fat mass
FM/FFM: Fat mass/fat-free mass ratio
FMI: Fat mass index
HE: Hand expressed
HM: Human milk
HMO: Human milk oligosaccharides
LCPUFA: Long-chain polyunsaturated fatty acids
M: Male
MeSH: Medical subject headings
N: Number of participants
n-3: Omega-3
n-6: Omega-6
NS: Not stated
PCA: Principal component analysis
PRISMA: Preferred Reporting Items for Systematic Reviews and Meta-Analysis
RCT: Randomized controlled trial
SD: Standard deviation
SDS: Standard deviation scores
SF: Skinfolds
