# Supplementary material for: Does human milk composition predict later risk of obesity? A systematic review
Source: BMC Nutr. 2023 Jul 20;9:89. doi: 10.1186/s40795-023-00742-9 (PMC10357724; doi:10.1186/s40795-023-00742-9)
Supplement: Supplementary file 2 — Additional file 2. Literature Search. Summary of all search terms and results. [file 40795_2023_742_MOESM2_ESM.docx]

**Table S2: Literature Search**

| Source: MEDLINE Ovid and Epub Ahead of Print, In-Process & Other Non-Indexed Citations (from 1946) | | |
| --- | --- | --- |
| Date Searched: 10 August 2022  Date Updated: 26 April 2023 | | |
| Line | **Terms** | **Records Found** |
| 1 | ((breast feeding or breastfeeding or breast milk or exclusively breastfed or breast fed or breast-fed or breastfed or human milk composition or human milk or human milk intake) not (complementary food* or breastfeeding duration or "complementary feed*")).tw,ot,hw. | 74793 |
| 2 | Milk, Human/an, ch [Analysis, Chemistry] | 9732 |
| 3 | 1 or 2 | 76530 |
| 4 | ((infant growth or anthropometry or body composition or infant body composition or weight or overweight or adiposity or obesity or p?diatric obesity or childhood obesity or obesity risk or weight-gain or growth velocity OR body mass index or BMI or child BMI) not (mental or cognitive or psychological or infection* or immun* or autoimmun*)).tw,ot,hw. | 1370522 |
| 5 | ((hormone* or cortisol or adiponectin or leptin or ghrelin or insulin or fat* or fatty acid* or omega-3* or essential fatty acid* or polyunsaturated fatty acid* or lipid* or alpha-linolenic acid or docosahexaenoic acid or eicosapentaenoic acid or omega-6* or gamma-linolenic acid or linoleic acid or carbohydrate* or lactose or fructose or glucose or human milk oligosaccharide* or oligosaccharide* or HMO* or amino acid* or protein* or whey or casein or energy intake or caloric intake or calorie*) not (microbiome or microbiota or gene* or genetic*)).tw,ot,hw. | 4773784 |
| 6 | ((adolescen* or child* or infan* or infant health) not (preterm or pre term or low birth weight or low birthweight or very low birthweight or very low birth weight or small for gestational age or SGA)).tw,ot,hw. | 4283694 |
| 7 | ((cohort or prospective birth or prospective cohort or prospective pilot or randomi?ed controlled trial or quasi-randomi?ed or cluster-randomi?ed) not (review or "systematic review")).tw,ot,hw. | 1333055 |
| 8 | 3 and 4 and 5 and 6 and 7 | 489 |
| 9 | limit 8 to humans | 469 |
| Date Updated: 26 April 2023 | | Additional papers found |
| 10 | limit 8 to (humans and yr="2022 - 2023") | 20 |

| Source: Embase Ovid (from 1974) | | |
| --- | --- | --- |
| Date Searched: 10 August 2022  Date Updated: 26 April 2023 | | |
| Line | **Terms** | **Records Found** |
| 1 | ((breast feeding or breastfeeding or breast milk or exclusively breastfed or breast fed or breast-fed or breastfed or human milk composition or human milk or human milk intake) not (complementary food* or breastfeeding duration or "complementary feed*")).tw,ot,hw. | 98281 |
| 2 | breast milk/ | 31325 |
| 3 | 1 or 2 | 99061 |
| 4 | ((infant growth or anthropometry or body composition or infant body composition or weight or overweight or adiposity or obesity or p?diatric obesity or childhood obesity or obesity risk or weight-gain or growth velocity or body mass index or BMI or child BMI) not (mental or cognitive or psychological or infection* or immun* or autoimmun*)).tw,ot,hw.  | 1861562 |
| 5 | ((hormone* or cortisol or adiponectin or leptin or ghrelin or insulin or fat* or fatty acid* or omega-3* or essential fatty acid* or polyunsaturated fatty acid* or lipid* or alpha-linolenic acid or docosahexaenoic acid or eicosapentaenoic acid or omega-6* or gamma-linolenic acid or linoleic acid or carbohydrate* or lactose or fructose or glucose or human milk oligosaccharide* or oligosaccharide* or HMO* or amino acid* or protein* or whey or casein or energy intake or caloric intake or calorie*) not (microbiome or microbiota or gene* or genetic*)).tw,ot,hw. | 5878539 |
| 6 | ((adolescen* or child* or infan* or infant health) not (preterm or pre term or low birth weight or low birthweight or very low birthweight or very low birth weight or small for gestational age or SGA)).tw,ot,hw. | 4050463 |
| 7 | ((cohort or prospective birth or prospective cohort or prospective pilot or randomi?ed controlled trial or quasi-randomi?ed or cluster-randomi?ed) not (review or "systematic review")).tw,ot,hw. | 1988502 |
| 8 | 3 and 4 and 5 and 6 and 7 | 636 |
| 9 | limit 8 to human | 626 |
| Date Updated: 26 April 2023 | | Additional papers found |
| 10 | limit 8 to (human and yr="2022 -Current") | 57 |

| Source: Maternity and Infant Care (MIDIRS) (from 1971) | | |
| --- | --- | --- |
| Date Searched: 10 August 2022  Date Updated: 26 April 2023 | | |
| Line | **Terms** | **Records Found** |
| 1 | ((breast feeding or breastfeeding or breast milk or exclusively breastfed or breast fed or breast-fed or breastfed or human milk composition or human milk or human milk intake) not (complementary food* or breastfeeding duration or "complementary feed*")).ab,hw,ti. | 24719 |
| 2 | (human - milk or Human milk).de. | 6 |
| 3 | 1 or 2 | 24719 |
| 4 | ((infant growth or anthropometry or body composition or infant body composition or weight or overweight or adiposity or obesity or p?ediatric obesity or childhood obesity or obesity risk or weight-gain or growth velocity or body mass index or BMI or child BMI) not (mental or cognitive or psychological or infection* or immun* or autoimmun*)).ab,hw,ti. | 34721 |
| 5 | ((hormone* or cortisol or adiponectin or leptin or ghrelin or insulin or fat* or fatty acid* or omega-3* or essential fatty acid* or polyunsaturated fatty acid* or lipid* or alpha-linolenic acid or docosahexaenoic acid or eicosapentaenoic acid or omega-6* or gamma-linolenic acid or linoleic acid or carbohydrate* or lactose or fructose or glucose or human milk oligosaccharide* or oligosaccharide* or HMO* or amino acid* or protein* or whey or casein or energy intake or caloric intake or calorie*) not (microbiome or microbiota or gene* or genetic*)).ab,hw,ti. | 24082 |
| 6 | ((adolescen* or child* or infan* or infant health) not (preterm or pre term or low birth weight or low birthweight or very low birthweight or very low birth weight or small for gestational age or SGA)).ab,hw,ti. | 117149 |
| 7 | ((cohort or prospective birth or prospective cohort or prospective pilot or randomi?ed controlled trial or quasi-randomi?ed or cluster-randomi?ed) not (review or "systematic review")).ab,hw,ti. | 33793 |
| 8 | 3 and 4 and 5 and 6 and 7 | 97 |
| Date Updated: 26 April 2023 | | Additional papers found |
| 9 | limit 8 to yr="2022 -Current" | 2 |

| Source: Web of Science Core Collection Clarivate (1990 onwards) | | | |
| --- | --- | --- | --- |
| Date Searched: 10 August 2022  Date Updated: 26 April 2023 | | | |
| Line | **Terms** | | **Records Found** |
| 1 | ALL=((("breast feeding" or breastfeeding or "breast milk" or "exclusively breastfed" or "breast fed" or "breast-fed" or breastfed or "human milk composition" or "human milk" or "human milk intake") not ("complementary food*" or "breastfeeding duration" or "complementary feed*") ) ) | | 69,134 |
| 2 | ALL=((("infant growth" or anthropometry or "body composition" or "infant body composition" or obes* OR "weight gain" OR overweight OR "over weight" or weight or adiposity or "p$ediatric obesity" or "childhood obesity" or "obesity risk" or "weight-gain" or "growth velocity" or "body mass index" or BMI or "child BMI") not (mental or cognitive or psychological or infection* or immun* or autoimmun*) ) ) | | 2,322,635 |
| 3 | ALL=(((hormone* or cortisol or adiponectin or leptin or ghrelin or insulin or fat* or "fatty acid*" or omega-3* or "essential fatty acid*" or "polyunsaturated fatty acid*" or lipid* or "alpha-linolenic acid" or "docosahexaenoic acid" or "eicosapentaenoic acid" or omega-6* or "gamma-linolenic acid" or "linoleic acid" or carbohydrate* or lactose or fructose or glucose or "human milk oligosaccharide*" or oligosaccharide* or HMO* or "amino acid*" or protein* or whey or casein or "energy intake" or "caloric intake" or calorie*) not (microbiome or microbiota or gene* or genetic*) ) ) | | 5,122,304 |
| 4 | ALL=(((adolescen* or child* or infan* or "infant health") not (preterm or "pre term" or "low birth weight" or "low birthweight" or "very low birthweight" or "very low birth weight" or "small for gestational age" or SGA) )) | | 3,782,481 |
| 5 | ALL=((cohort or "prospective birth" or "prospective cohort" or "prospective pilot" or "randomi?ed controlled trial" or "quasi-randomi$ed" or "cluster-randomi$ed") not (review or "systematic review")) | | 938,890 |
| 6 | #5  AND  #4  AND  #3  AND  #2  AND  #1 | | 356 |
| Date Updated: 26 April 2023 | | Additional papers found | |
| 7 | #6 AND #5 AND #4 AND #3 AND #2 AND #1 AND PY=(2022-2023) | | 19 |

| Source: SCOPUS Elsevier (1979 onwards) | | |
| --- | --- | --- |
| Date Searched: 10 August 2022  Date Updated: 26 April 2023 | | |
| Line | **Terms** | **Records Found** |
| 1 | TITLE-ABS-KEY ( ( {breast feeding} OR breastfeeding OR {breast milk} OR {exclusively breastfed} OR {breast fed} OR {breast-fed} OR breastfed OR {human milk composition} OR {human milk} OR {human milk intake} ) AND NOT ( {complementary food*} OR {breastfeeding duration} OR {complementary feed*} ) ) | 102205 |
| 2 | TITLE-ABS-KEY ( ( {infant growth} OR anthropometry OR {body composition} OR {infant body composition} OR obes* OR {weight gain} OR overweight OR {over weight} OR weight OR adiposity OR {p?ediatric obesity} OR {childhood obesity} OR {obesity risk} OR {weight-gain} OR {growth velocity} OR {body mass index} OR bmi OR {child BMI} ) AND NOT ( mental OR cognitive OR psychological OR infection* OR immun* OR autoimmun* ) ) | 2,848,429 |
| 3 | TITLE-ABS-KEY ( ( hormone* OR cortisol OR adiponectin OR leptin OR ghrelin OR insulin OR fat* OR "fatty acid*" OR {omega-3} OR "essential fatty acid*" OR "polyunsaturated fatty acid*" OR lipid* OR {alpha-linolenic acid} OR {docosahexaenoic acid} OR {eicosapentaenoic acid} OR {omega-6} OR {gamma-linolenic acid} OR {linoleic acid} OR "carbohydrate*" OR lactose OR fructose OR glucose OR "human milk oligosaccharide*" OR "oligosaccharide*" OR "HMO*" OR "amino acid*" OR "protein*" OR whey OR casein OR {energy intake} OR {caloric intake} OR "calorie*" ) AND NOT ( microbiome OR microbiota OR "gene*" OR "genetic*" ) ) | 6,838,385 |
| 4 | TITLE-ABS-KEY ( ( "adolescen*" OR "child*" OR "infan*" OR {infant health} ) AND NOT ( preterm OR {pre term} OR {low birth weight} OR {low birthweight} OR {very low birthweight} OR {very low birth weight} OR {small for gestational age} OR sga ) ) | 5,317,212 |
| 5 | TITLE-ABS-KEY ( ( cohort OR {prospective birth} OR {prospective cohort} OR {prospective pilot} OR {randomi?ed controlled trial} OR {quasi-randomi?ed} OR {cluster-randomi$ed} ) AND NOT ( review OR {systematic review} ) ) | 945,946 |
| 6 | 1 AND 2 AND 3 AND 4 AND 5 | 406 |
| Date Updated: 26 April 2023 | | Additional papers found |
| 7 | 6 AND ( LIMIT-TO ( PUBYEAR , 2023 ) OR LIMIT-TO ( PUBYEAR , 2022 ) ) | 41 |

| Source: PubMed | | |
| --- | --- | --- |
| Date Searched: 10 August 2022  Date Updated: 26 April 2023 | | |
| Line | **Terms** | **Records Found** |
| 1 | (((((((cohort or prospective birth or prospective cohort or prospective pilot or randomi?ed controlled trial or quasi-randomi?ed or cluster-randomi?ed) not (review or "systematic review"))) AND (((cohort or prospective birth or prospective cohort or prospective pilot or random?ed controlled trial or quasi random?ed or cluster random?ed) not (review or "systematic review")))) AND (((adolescen* or child* or infan* or infant health) not (preterm or pre term or low birth weight or low birthweight or very low birthweight or very low birth weight or small for gestational age or SGA)))) AND (((hormone* or cortisol or adiponectin or leptin or ghrelin or insulin or fat or fatty acid* or omega-3 or essential fatty acid* or polyunsaturated fatty acid* or lipid* or alpha-linolenic acid or docosahexaenoic acid or eicosapentaenoic acid or omega-6 or gamma-linolenic acid or linoleic acid or carbohydrate* or lactose or fructose or glucose or human milk oligosaccharide* or oligosaccharide* or HMO or amino acid* or protein* or whey or casein or energy intake or caloric intake or calorie*) not (microbiome or microbiota or gene* or genetic)))) AND (((infant growth or anthropometry or body composition or infant body composition or weight or overweight or adiposity or obesity or p?diatric obesity or childhood obesity or obesity risk or weight-gain or growth velocity, body mass index or BMI or child BMI) not (mental or cognitive or psychological or infection* or immun* or autoimmun*)))) AND ((human milk[MeSH Major Topic]) AND (((breast feeding or breastfeeding or breast milk or exclusively breastfed or breast fed or breast-fed or breastfed or human milk composition or human milk or human milk intake) not (complementary food* or breastfeeding duration or "complementary feed*")))) | 127 |
| Date Updated: 26 April 2023 | | Additional papers found |
| 2 | Filters: from 2022 - 2023 | 1 |

| Source: Cochrane Central Register of Controlled Trials (CENTRAL; current issue) part of the Cochrane Library | | |
| --- | --- | --- |
| Date Searched: 10 August 2022  Date Updated: 26 April 2023 | | |
| Line | **Terms** | **Records Found** |
| 1 | (breastfeeding):ti,ab,kw | 6565 |
| 2 | MeSH descriptor: [Milk, Human] explode all trees | 1138 |
| 3 | ((breast feeding or breastfeeding or breast milk or exclusively breastfed or breast fed or breast-fed or breastfed or human milk composition or human milk or human milk intake) not (complementary food* or breastfeeding duration or "complementary feed*")) | 13430 |
| 4 | ((infant growth or anthropometry or body composition or infant body composition or weight or overweight or adiposity or obesity or p?diatric obesity or childhood obesity or obesity risk or weight-gain or growth velocity, body mass index or BMI or child BMI) not (mental or cognitive or psychological or infection* or immun* or autoimmun*)) | 174428 |
| 5 | ((fatty acid* or omega-3 or essential fatty acid* or polyunsaturated fatty acid* or alpha-linolenic acid or docosahexaenoic acid or eicosapentaenoic acid or omega-6 or gamma-linolenic acid or linoleic acid) not (microbiome or microbiota or gene* or genetic)) | 17530 |
| 6 | #1 AND #2 AND #3 AND #4 AND #5 | 7 |
| Date Updated: 26 April 2023 | | Additional papers found |
| 7 | Limit publication year to 2022 and 2023 | 0 |

| Source: Citation Search | | |
| --- | --- | --- |
| Last Date Searched: 10 August 2022 | | |
| Source | **Paper found** |  |
| Ramadurai et al. 2022 [11] | Ellsworth et al. 2020 [29] |  |
| Galante et al. 2020 [17] | Zamanillo et al. (2019) [16] |  |
| Brunner et al. 2015 [28] | Miralles et al. 2006 [33] |  |
| Gridneva et al. 2019 [31] | Gridneva et al. 2018 [30] |  |
| Zhang et al. 2021 [47] | Lind et al. 2018 [10] |  |
|  | Eriksen et al. 2018 [21] |  |
|  | Jorgensen et al. 2021 [15] |  |
